# Factors Influencing Energy Drink Consumption in Participants and Viewers of Extreme Sports

**DOI:** 10.1155/2020/9382521

**Published:** 2020-10-07

**Authors:** Conrad A. Goodhew, Tracy L. Perry, Nancy J. Rehrer

**Affiliations:** ^1^Department of Human Nutrition, University of Otago, P.O. Box 56, Dunedin 9054, New Zealand; ^2^Division of Sciences, University of Otago, P.O. Box 56, Dunedin 9054, New Zealand; ^3^School of Physical Education Sport & Exercise University of Otago, P.O. Box 56, Dunedin 9054, New Zealand

## Abstract

**Objective:**

To quantify energy drink consumption and influences affecting consumption in those who participate in or watch extreme sports.

**Methods:**

An online survey, informed by focus groups, was administered via Quadrics®. Advertisement was via social media, emailing extreme sport clubs, flyers at extreme sport locations, and word of mouth. Participation was limited to those >18 y who watched and/or participated in extreme sports. The study was conducted in New Zealand, with international online availability. Variables measured comprised age, sex, energy drink consumption, reasons for their use, extreme sport viewing, advertising, and sponsorship. Logistic regression models were utilised.

**Results:**

Amongst participants who completed the questionnaire (*n* = 247), the mean (SD) age was 26.2 (8.2) y, 40.5% were female, 57.9% consumed energy drinks, and 25.5% consumed >one per week. For every year older, odds of consuming energy drinks were 3.1% lower (*p*=0.04). A 31% increase in energy drink consumption for every single increase of viewing extreme sport per week was observed (*p*=0.009); however, reported viewing of advertising was not associated with increased consumption.

**Conclusions:**

A large proportion of extreme sport enthusiasts regularly consume energy drinks, especially younger adults. Extreme sport viewing, where energy drink sponsorship is common, appears to increase their consumption, even if not considered advertising by the viewers themselves.

## 1. Introduction

Extreme sport participation is on the rise and becoming more popular than traditional sports [1–3]. With an increased ability to view extreme sports online through YouTube® and social media, access to these sports is readily available. Extreme sports are considered unconventional, thereby creating an opportunity for energy drink manufacturers to align themselves with these sports [4]. Although sponsorship links energy drinks with extreme sports, it is unknown whether the viewing of or participation in extreme sports influences the consumption of these beverages.

There is no universally accepted definition of extreme sports [5]. For our study, we have defined extreme sports as sports “in which a physical thrill, commonly known as an adrenaline rush, often takes place, and physical and mental challenges occur frequently requiring fast and accurate reactions, and in which a mistake or accident may result in lifelong disability or death.” An energy drink was defined as “a carbonated beverage that contains caffeine and other ingredients to increase the perceived energy and vitality for the consumer” [6, 7].

There are no current upper limits or restrictions on caffeine; however, adverse effects have been found with consumption greater than 400 mg per day, known as caffeine intoxication [8, 9]. Effects of caffeine intoxication include anxiety, sleeplessness, insomnia, diuresis, gastrointestinal upset, increased heart rate and blood pressure, tremors, tachycardia, and even death [10, 11]. Several fatal heart attacks related to excessive caffeine ingestion from energy drinks have been reported [12–16], indicating a possible health risk when consumed in excess.

Reported energy drink consumption rates within those >18 y range from 3% to 60%, varying by population and manner of assessment [17–20], with consumption rates having dramatically increased over the last two decades [11, 20]. Questionnaire-based surveys reported that university students who consumed energy drinks within the last 30 days, or on average one per month, range from 11% to 70% [6, 21–28]. Furthermore, high energy drink consumers, who drink ≥1 per week, or 6 in the last 30 days, range from 10% to 59% [6, 21–27]. Uzundumlu et al. [28] found that the main motivators for energy drink consumption were a lack of sleep, to increase energy, and preparing for stressful events, such as exams or major deadlines. Others found that participants who consumed ≥1 energy drink per week (or ≥4 per month) were more likely to participate in risk-taking behaviours, such as extreme sports [29–31].

A cross-sectional analysis of 2,287 young adults (45% male, mean age 25.3 y) that included assessment of energy drink consumption and influences [29] found that males who had high energy drink consumption were more physically active, but were also more likely to have muscle-enhancing behaviours including substance use. They also found that females who had high energy drink consumption were more likely to diet and use marijuana. The authors considered these risk-taking behaviours and noted that similar patterns occurred in those with a high enjoyment of participating in sensation-seeking behaviours, i.e., “doing things that are more than a little dangerous” [29].

Miller [30] surveyed 602 undergraduate students and found that high energy drink consumption was significantly correlated with participation in extreme sports. Spierer et al. [31] also found high energy drink consumption was related to an increased participation in extreme sports, as well as partaking in other risky behaviours—such as substance abuse and drink driving.

To our knowledge, there is no published research on energy drink consumption and effects of sponsorship and advertising amongst participants or followers of extreme sports. The purpose of this study was to determine (1) consumption rates of energy drinks, (2) reasons for consuming or not consuming energy drinks, and (3) possible correlations between external motivators, including advertising and sponsorship, and energy drink consumption, in extreme sport enthusiasts.

## 2. Materials and Methods

Ethical approval was granted by the University of Otago Human Ethics Committee (Health), after evidence of peer review of protocol and written submission of application (D16/365).

### 2.1. Questionnaire

Questions from previous surveys [6, 19, 21, 22, 32] on energy drink use were reviewed and adapted for the target audience. Focus groups were then conducted to refine the questionnaire.

Seven extreme sport enthusiasts (18–24 y) were recruited to pretest the 35-question survey. They were given the Qualtrics® questionnaire 24 hours prior to a one-hour focus group. The question appraisal system for focus group pretesting (QAS-2009) was used to ensure the respondents' answers aligned with their internally generated answers [33].

The final questionnaire comprised 41 questions. By using skip and display logic, respondents were able to answer only those questions that were relevant to them based on their previous answers. Online information sheets, a consent form, and prize draw contact information were included prior to accessing the questionnaire.

### 2.2. Recruitment and Inclusion Criteria

The online questionnaire was live between November 2016 and February 2017. Participation was voluntary, and anonymous recruitment was conducted throughout this time via weekly social media posts, emails to major extreme sport clubs, flyers at extreme sport events and locations, and word of mouth. Randomly drawn prizes offered as an incentive included an AJ Hackett International Bungy Jump, Nitro Circus VIP Tickets, and an Amazon Voucher, via a separate link to ensure survey answers remained anonymous.

Only respondents who were ≥18 y at the time of survey completion and indicated that they followed and or participated in extreme sport(s) were included. At least 84% of the survey was required to be completed for the respondent's answers to be included in the analysis. Respondents' data were excluded from analysis if the sport they followed and or participated in was not considered extreme, e.g., horse riding. This was at the discretion of the authors.

### 2.3. Data Analysis

Descriptive analytics were obtained via cross-tabulation software in Qualtrics®. Percentages were calculated by taking the number of respondents that clicked on an answer and dividing this by the number of respondents that answered that question, regardless of whether a respondent was able to choose one or more answers. Logistic regression models were generated using Stata 14.2 (StataCorp, Texas) for differences in energy drink consumption relating to age, gender, frequency of extreme sport viewing, and advertising. All statistical comparisons were 2-tailed, and *p* values <0.05 were considered statistically significant.

## 3. Results

### 3.1. Participants

Two hundred and fifty two surveys were completed, and 247 were included in the analyses. Five respondents were removed as they either indicated a sports drink as an energy drink (*n* = 2), listed sports that were not extreme (*n* = 2), or completed the survey twice (*n* = 1). Respondent demographics are displayed in Table 1. The mean (SD) age was 26.2 (8.2) y, and the majority had completed education beyond secondary school (*n* = 209/246, 85%). Of the respondents, 137 (55.5%) participated in extreme sports, 229 (92.7%) followed extreme sports, and 119 (48.2%) had both participated and followed extreme sports.

### 3.2. Energy Drink Consumption

**Figure 1:**
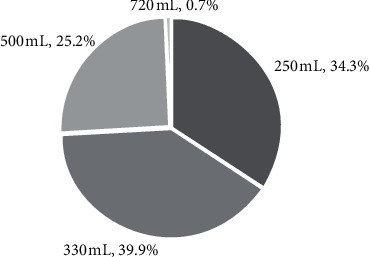
Proportion of energy drink consumers ingesting a given volume of energy drink at one time.

Amongst those surveyed, 57.9% (*n* = 143) consumed energy drinks with 25.5% (*n* = 63) consuming ≥1 energy drink per week. A greater proportion of males surveyed (*n* = 89, 60.5%) consumed energy drinks than females (*n* = 54, 54.0%), but this was not significant (*p*=0.31). Age also influenced energy drink consumption; for every year older, the odds of consumption were 3.1% lower (OR 0.969; 95% confidence interval [CI]: 0.941 to 0.999; *p*=0.04). The most commonly stated reasons for avoiding energy drinks were that they were perceived as unhealthy, to avoid caffeine, and that they did not like the taste (Table 2). The most commonly stated reasons for consuming energy drinks were because it “helped keep them awake,” that they needed “a lift” or “get up and go,” and for refreshment or taste (Table 3). Commonly stated side effects of energy drinks included sleep disturbance and being “jittery” or “shaking” (Table 4). Of those that consumed energy drinks, 65.7% (*n* = 94) reported consuming servings larger than one standard energy drink in a single sitting (250 mL, ∼90 mg of caffeine, Figure 1). Furthermore, 43.0% (*n* = 107) of those that consumed energy drinks indicated a brand preference. Most respondents (86.0%) chose the particular brand of energy drink because of the taste, but only 13.1% consumed energy drinks because the brand sponsored an extreme sport that they participated in, and 2.8% because their favourite sports person was sponsored by it. Surprisingly, the two respondents who were sponsored by an energy drink company did not consume energy drinks.

### 3.3. Extreme Sports and Energy Drink Consumption

**Figure 2:**
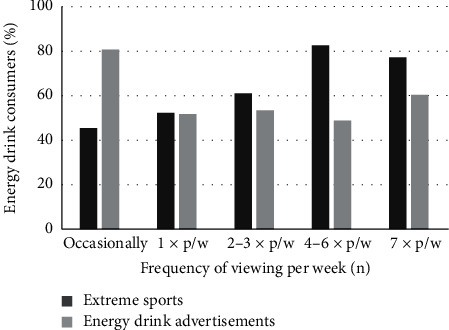
Proportional frequency of viewing of extreme sports and energy drink advertisements by energy drink consumers.

There was a positive association between the frequency of extreme sport viewing and energy drink consumption (Figure 2). The odds of a respondent consuming energy drinks were 31% greater for every single increase of viewing extreme sport per week (OR 1.31; 95% CI: 1.13 to 1.52; *p*=0.009). There was no association between energy drink consumption and stated viewing of advertising in those that consumed energy drinks (OR 0.978; 95% CI: 0.88 to 1.08; *p*=0.11). However, the odds of a respondent who indicated that they consumed a large number of energy drinks (≥1 energy drink per week) increased 14% for every increase in viewing one energy drink advertisement per week (OR 1.14; 95% CI: 1.01 to 1.30; *p*=0.04). Furthermore, two-thirds of respondents who consumed energy drinks and participated in extreme sports indicated that they got into the sport because they were “brought up with the sport” and/or “had a close family or friend” who participated.

Of the respondents who watched extreme sports, 92.1% did so on the Internet (including YouTube®, social media, and websites other than social media). Only 67% and 25% stated that they followed extreme sports on television or went to live events, respectively. Energy drink advertising was most frequently viewed through social media, websites, and YouTube® (81.3%, *n* = 200/246). Advertising of energy drinks was also observed at local shops (76.8%), while watching extreme sport (72.8%), and on television (66.3%).

## 4. Discussion

Energy drinks are prevalent within the extreme sport subculture due to sponsorship of events and athletes. Events have become more accessible with multimedia platforms for viewing increasing in number. To our knowledge, this is the first survey to include questions that examined extreme sport enthusiasts' energy drink consumption and motivators.

In a survey of New Zealand high school students, 35% had consumed energy drink(s), with 12% having consumed ≥4 within the past week [34]. Amongst Australian adolescents, 56% professed as being “lifetime” users with 36% having consumed more than 2/day [35]. In Canada, in a large sample amongst 12–24 year olds, of those who chose to answer questions on energy drinks, 74% reported “ever” consuming energy drinks, with 16% having consumed one in the last week [36] with 12% of surveyed 12–20 year olds in another Canadian study having consumed at least one in the last week [37]. Amongst students of a university in the USA, 59% consumed at least one energy drink in the last seven days and 64% consumed ≥1/week in the last month [26]. Amongst Argentinian physical education students >21 years of age, 65% had used energy drinks at least once and 39% had used six or more times in the last month [23]. That study, as well as those of Pettit and DeBarr [26] and Ballistreri and Corradi-Webster [23], had similar or higher consumption than that observed in the current study; however, theirs were with a younger demographic. In the current study, for every year older, a respondent was 3.1% less likely to consume energy drinks. This supports the findings of Pennay et al. [19] that younger individuals (18–24 y) were more likely to consume energy drinks than the older (>25 y).

Previous studies have reported that males were more likely to consume energy drinks than females [19, 27, 28, 34] and that males were also more likely to have risk-taking tendencies [19, 38]. In the present study, however, there was no difference in energy drink consumption between the sexes. It is possible that extreme sport enthusiasts are considered risk takers, regardless of whether they are male or female.

It is also interesting that the two respondents who were sponsored by an energy drink company did not consume energy drinks. It appears, thus, that age is a stronger predictor of energy drink consumption than sex and that direct sponsorship may not influence consumption by the individual being sponsored.

The top reasons given for consuming energy drinks were to “help keep me awake” or “need a lift” or “get up and go.” Malinauskas et al. [25] also investigated reasons for consumption and found that university students consumed energy drinks to increase energy, which was consistent with our finding. In the present study, refreshment and taste were strong drivers for energy drink consumption. This finding was similar to that of Arpaci et al. [21] in which it was found that 67% of those that consumed energy drinks did so for taste. This could be problematic for any health initiatives and education concerning energy drinks, as Verbeke [39] found that consumers were less likely to sacrifice the taste of food and beverages for a healthy alternative, indicating that taste is a strong effecter of suboptimal food or beverage consumption.

In our study, the most commonly stated reasons for avoiding energy drinks were because they were perceived as unhealthy, to avoid caffeine, or because they did not like the taste. Few studies have investigated the reasons why individuals do not consume energy drinks. However, Aslam et al. [22] found that “no specific reason” and “awareness of side effects” were the main reasons for medical students to avoid consuming energy drinks. We can conclude that there are multiple reasons why individuals avoid energy drinks.

It was hypothesised that energy drink advertisement exposure would increase consumption rates, but surprisingly, in the present study, no correlation between overall energy drink consumption and reported viewing of advertising was observed. However, the frequency of watching extreme sports was positively correlated with consumption. Thus, it was not the explicit advertising that the individual was aware of that affected consumption, but rather that there may have been some unconscious influence on behaviour. Watching those sports in which individuals and the sports themselves are frequently sponsored by energy drinks, with branding on clothing and equipment used during completion, although not seen as advertising, may be influencing behaviour. In fact, in the present study, every increase per week in extreme sport viewing increased the odds of energy drink consumption. In addition, participants who were “brought up with the sport” were more likely to consume energy drinks, whereby long-term exposure would occur. This is supported by Bustin et al. [40], who found that individuals with thrill-seeking tendencies were exposed to subliminal advertising by Red Bull® and were more likely to consume energy drinks. The increased exposure, with increased viewing of events in which the events themselves and competitors are sponsored, appears to increase the likelihood of consumption in these risk-taking individuals.

## 5. Conclusions

In this demographic, those who participate in and/or watch extreme sports consume large-sized energy drinks on a regular basis. The predominant reasons given for their consumption were for stimulatory effects and taste. Interestingly, this demographic did not think that advertisement influenced their consumption habits, and only a small proportion stated that sponsorship of a sport or a person influenced their consumption. Nevertheless, increased viewing of extreme sports, where energy drink sponsorship is commonplace, was associated with increased consumption. Viewing of these sports occurred primarily via social media and other online applications. Thus, it appears that a more nuanced form and venue of marketing is increasing energy drink consumption in this young adult, predominantly male, population. Our findings warrant further investigation to understand a demographic that is susceptible to influences that affect consumption behaviours and may lead to increased sugar and caffeine intake.

## Figures and Tables

**Table 1:** Demographics of those completing the questionnaire on energy drink consumption.

Category	*n*	(%)
Sex		
Male	147	59.5
Female	100	40.5

Age (y)		
18–24	147	59.5
25–34	65	26.3
35–49	27	10.9
50+	8	3.2

Education^a^		
Completed secondary education	131	53.3
Completed undergraduate (or higher) degree or diploma	108	43.9

Occupation^b^		
Paid employment	175	71.3
Student	88	35.6
Unemployed	9	3.6
Professional athlete	6	2.4

Ethnicity^b^		
NZ/AUS European	164	66.4
European (other than above)	59	23.8
Maori	29	11.7
Pacific Islander	14	5.7

Extreme sports participated in^b,c^		
Snow sports	72	52.6
Nonmotor sports	68	49.6
Water sports	67	48.9
Air sports	43	31.4
Motor sports	37	27.0
Ultimate fight club	7	5.1

Extreme sports followed^b,c^		
Snow sports	113	49.3
Nonmotor sports	100	43.7
Water sports	121	52.8
Air sports	77	33.6
Motor sports	140	61.1
Ultimate fight club	90	39.3

^a^Those included in the ‘completed secondary education' had completed high school and/or some postsecondary education but had not completed an undergraduate degree or higher. ^b^Respondent could choose multiple answers. ^c^Categorised extreme sports from a total of 42 options plus “other (please specify).”

**Table 2:** Why respondents consume energy drinks (*n* = 143)^a^.

Reasons given	*n*	(%)
Helps to keep me awake	74	51.7
Need a lift or get up and go	72	50.3
Refreshment/taste	68	47.6
Helps with long-distance driving	52	36.4
When partying	50	35.0
Increases energy for physical activity or sport	45	31.5
Aids you to study or complete a major project	41	28.7
I get it for free	19	13.3
Stress relief	13	9.1
Coffee replacement^b^	2	1.4
Addiction^b^	2	1.4
For weight loss purposes	1	0.7
It was advertised	1	0.7

^a^Respondent could choose multiple answers. ^b^Indicated in the “other (please specify)” section.

**Table 3:** Why respondents avoid energy drinks (*n* = 104)^a^.

Reasons given	*n*	(%)
Perceived as unhealthy^b^	42	40.4
I try to avoid caffeine in general	39	37.5
I do not like the taste	36	34.6
Weight gain	30	28.8
I do not like the feeling it creates after consumption	23	22.1
I lose sleep when I use it	17	16.3
It makes me shake and/or tingle	13	12.5
It makes me dehydrated	9	8.7
I get headaches after consumption	8	7.7
It gives me vomiting, nausea, and/or abdominal pain	3	2.9
I get chest pains and/or heart problems after consumption	3	2.9
Costly	2	1.9

^a^Respondent could choose multiple answers. ^b^Indicated in the “other (please specify)” section.

**Table 4:** Side effects of energy drinks amongst consumers (*n* = 143)^a^.

Side effect	*n*	(%)
Sleep disturbance	65	45.8
Jittery or shaking	58	40.8
Dehydration	34	23.9
Headaches	29	20.4
Fatigue	26	18.3
Have trouble focusing on tasks	15	10.6
Vomiting, nausea, and abdominal pain	10	7.0
Reduced accuracy of movement	6	4.2
Muscle stiffness and aches	2	1.4
Increased heart rate^b^	1	0.7
No adverse effects	47	33.1

^a^Respondent could choose multiple answers. ^b^Indicated in the “other (please specify)” section.

## Data Availability

Most of the raw data are included in the article. Any data not included can be made available upon request from the corresponding author.

## References

[1] Puchan H. (2004). Living “extreme”: adventure sports, media and commercialisation. *Journal of Communication Management*.

[2] Thorpe H., Wheaton B. (2011). “Generation X games”, action sports and the olympic movement: understanding the cultural politics of incorporation. *Sociology*.

[3] Thorpe H., Wheaton B., Thorpe H. A. (2017). The X games: re-imagining youth and sport. *Sport, Media and Mega-Events*.

[4] Jung Woo L. (2015). The meaning of sport: sociolinguistic analysis of sport and energy drink brands’ advertising messages. *International Journal of Sport Communication*.

[5] Cohen R. (2012). The relationship between personality, sensation seeking, reaction time and sport participation: evidence from drag racers, sport science students and archers. *School of Health and Social Sciences*.

[6] Alsunni A. A., Badar A. (2011). Energy drinks consumption pattern, perceived benefits and associated adverse effects amongst students of University of Dammam, Saudi Arabia. *Journal Ayub Med Coll Abbottabad*.

[7] Mead A. (2001). Extreme sports: energy drinks. *Sport Health*.

[8] Heckman M. A., Sherry K., De Mejia E. G. (2010). Energy drinks: an assessment of their market size, consumer demographics, ingredient profile, functionality, and regulations in the United States. *Comprehensive Reviews in Food Science and Food Safety*.

[9] Mitchell D. C., Knight C. A., Hockenberry J., Teplansky R., Hartman T. J. (2014). Beverage caffeine intakes in the USA. *Food and Chemical Toxicology*.

[10] Kerrigan S., Lindsey T. (2005). Fatal caffeine overdose: two case reports. *Forensic Science International*.

[11] Reissig C. J., Strain E. C., Griffiths R. R. (2009). Caffeinated energy drinks--a growing problem. *Drug Alcohol Depend*.

[12] Berger A. J., Alford K. (2009). Cardiac arrest in a young man following excess consumption of caffeinated “energy drinks”. *Medical Journal of Australia*.

[13] Farverov S. (2014). *“Energy Drinks Blamed for Sixteen-Year-Old High School Softball Star’s Fatal Heart Attack on Mexican Beach Vacation”*.

[14] Kydo (2015). *“Japanese Man Dies after Daily Heavy Consumption of Caffeinated Beverages”*.

[15] Quinn L. (2017). *“Healthy Student, 16, Collapses and Dies from Heart Problems after Drinking a Large Mountain Dew, a Latte and an Energy Drink in the Space of Two Hours”*.

[16] Newshub (2017). *“Monster Energy Sued over Teen’s Death”*.

[17] Alsunni A. A. (2015). Energy drink consumption: beneficial and adverse health effects. *International Journal of Health Sciences*.

[18] Peacock A., Droste N., Pennay A., Miller P., Lubman D. I., Bruno R. (2015). Awareness of energy drink intake guidelines and associated consumption practices: a cross-sectional study. *BMC Public Health*.

[19] Pennay A., Cheetham A., Droste N. (2015). An examination of the prevalence, consumer profiles, and patterns of energy drink use, with and without alcohol, in Australia. *Alcoholism: Clinical and Experimental Research*.

[20] Thomson B. M., Campbell D. M., Cressey P., Egan U., Horn B. (2014). Energy drink consumption and impact on caffeine risk. *Food Additives & Contaminants: Part A*.

[21] Arpaci N., Tosun S., Ersoy G. (2010). Sports and energy drink consumption of physical education & sports students’ and their knowledge about them. *Ovidius University Annals, Series Physical Education & Sport/Science, Movement & Health*.

[22] Aslam H. M., Mughal A., Edhi M. M. (2013). Assessment of pattern for consumption and awareness regarding energy drinks among medical students. *Archives of Public Health*.

[23] Ballistreri M. C., Corradi-Webster C. M. (2008). Consumption of energy drinks among physical education students. *Revista Latino-Americana de Enfermagem*.

[24] Gallucci A. R., Martin R. J., Morgan G. B. (2016). The consumption of energy drinks among a sample of college students and college student athletes. *Journal of Community Health*.

[25] Malinauskas B. M., Aeby V. G., Overton R. F., Carpenter-Aeby T., Barber-Heidal K. (2007). A survey of energy drink consumption patterns among college students. *Nutrition Journal*.

[26] Pettit M. L., DeBarr K. A. (2011). Perceived stress, energy drink consumption, and academic performance among college students. *Journal of American College Health*.

[27] Reid S. D., Ramsarran J., Brathwaite R. (2015). Energy drink usage among university students in a Caribbean country: patterns of use and adverse effects. *Journal of Epidemiology and Global Health*.

[28] Uzundumlu A. S., Sezgin A., Sari M. M. (2016). Analysis of factors affecting the status of energy drink usage status by university students. *Studies on Ethno-Medicine*.

[29] Larson N., Laska M. N., Story M., Neumark-Sztainer D. (2015). Sports and energy drink consumption are linked to health-risk behaviours among young adults. *Public Health Nutrition*.

[30] Miller K. E. (2008). Energy drinks, race, and problem behaviors among college students. *Journal of Adolescent Health*.

[31] Spierer D. K., Blanding N., Santella A. (2014). Energy drink consumption and associated health behaviors among university students in an urban setting. *Journal of Community Health*.

[32] Murad H. A. S., Rafeeq M. M. (2016). Pattern of use and awareness of contents, benefits and adverse effects of energy drinks among university students in Rabigh, Saudi Arabia. *Biomedical Research (India)*.

[33] Willis G. B. (2015). *Analysis of the Cognitive Interview in Questionnaire Design*.

[34] Utter J., Denny S., Teevale T., Sheridan J. (2018). Energy drink consumption among New Zealand adolescents: associations with mental health, health risk behaviours and body size. *Journal of Paediatrics and Child Health*.

[35] Costa B. M., Hayley A., Miller P. (2016). Adolescent energy drink consumption: an Australian perspective. *Appetite*.

[36] Reid J. L., McCrory C., White C. M. (2017). Consumption of caffeinated energy drinks among youth and young adults in Canada. *Preventive Medicine Reports*.

[37] Sampasa-Kanyinga H., Hamilton H. A., Chaput J.-P. (2017). Sleep duration and consumption of sugar-sweetened beverages and energy drinks among adolescents. *Nutrition*.

[38] Wimer D. J., Levant R. F. (2013). Energy drink use and its relationship to masculinity, jock identity, and fraternity membership among men. *American Journal of Men’s Health*.

[39] Verbeke W. (2006). Functional foods: consumer willingness to compromise on taste for health?. *Food Quality and Preference*.

[40] Bustin G. M., Jones D. N., Hansenne M., Quoidbach J. (2015). Who does red bull give wings to? sensation seeking moderates sensitivity to subliminal advertisement. *Frontiers in Psychology*.

